# Depression and the Risk Factors Among Elderly Residents in Gyeongsangbuk-Do

**DOI:** 10.1192/bjo.2024.191

**Published:** 2024-08-01

**Authors:** M.D. Kwang Hun Lee

**Affiliations:** Dongguk University Hospital, Gyeongju, Republic of Korea

## Abstract

**Aims:**

The purpose of this study is to analyze the prevalence and factors of depression among the elderly population, a significant issue in Korea's aging society. By doing so, we aim to provide basic indicators for improving mental health and quality of life while efficiently managing healthcare costs.

**Methods:**

From February to December 2021, a study was conducted on a population of 19,158 elderly individuals aged 65 and above residing in Gyeongsangbuk-do province. The severity of depression was evaluated using the Korean version of the Patient Health Questionnaire (PHQ)-9, which was adapted for use as a depression screening tool in clinical settings. In addition, demographic information such as place of residence, age, gender, and education level was collected to analyze factors that may influence depression. The data were analyzed using cross-analysis, two independent sample t-tests, one-way ANOVA, multiple regression analysis, and Scheffe's post-hoc analysis.

**Results:**

In the PHQ-9 screening, the average score of the elderly population was 3.65. The results showed that 13,705 individuals (71.5%) were in the normal group with scores ranging from 0 to 4, 3,683 individuals (19.2%) were in the mild group with scores ranging from 5 to 9, 1,575 individuals (8.2%) were in the moderate group with scores ranging from 10 to 19, and 195 individuals (1.0%) were in the severe group with scores of 20 or higher. It was found that place of residence, education level, type of housing, top two difficulties in daily life, subjective economic status, desired services, subjective mental health, past and current history of mental health treatment, and medication for physical illness had statistically significant (*p < 0.05) effects on depression.

**Conclusion:**

Various factors were found to have a significant impact on depression among the elderly population in Gyeongsangbuk-do. Proactive prevention and treatment tailored to the population characteristics of the region may be necessary.

